# Herbal Therapeutics in Alzheimer's Disease: Investigating Behavioral and Biochemical Changes in Animal Model

**DOI:** 10.1002/alz70858_097103

**Published:** 2025-12-24

**Authors:** Saara Ahmad Muddasir Khan, Asra Khan, Ayesha Siddiqua, Muhammad Wasim, Sumaiya Binte Hamid, Saiqa Tabassum

**Affiliations:** ^1^ The Aga Khan University, Karachi, Pakistan; ^2^ Department of Pathology and Pathophysiology, School of Basic Medical Sciences, College of Medicine, Zhengzhou University, Zhengzhou, Henan, 450001, China, Zhengzhou, Henan, China; ^3^ The Aga Khan University, KARACHI, Pakistan; ^4^ Maternal and Children's Health Research Institute, Shunde Women and Children's Hospital, Guangdong Medical University, Foshan, 528300 China, China, Pakistan; ^5^ Aga Khan University, Karachi, Pakistan; ^6^ Szabist, Karachi, Pakistan

## Abstract

**Background:**

Alzheimer disease (AD) is a progressive neurodegenerative disorder that gradually leads to cognitive decline and memory impairment Standard therapies like Donepezil and Memantine help alleviate symptoms but their effect on the advancement of disease is restricted. Natural compounds such as Carum *Copticum* Benth (Ajwain) and its active constituent Thymol, exhibit neuroprotective and cognitive enhancement effects indicating a potential therapeutic effect on AD. This research provides a comparative effectiveness evaluation on the application of Carum *Copticum* and Thyme oil (Thymol, primarily) with or without standard treatments in the AD‐like model mice.

**Method:**

We created a model of AD in rats with aluminium chloride (AlCl3) and D‐galactose. There were six groups, Healthy Control, Diseased Control (AD), Donepezil‐, Memantine‐, Carum *Copticum*‐ and Carum *Copticum* + Thymol‐treated. A series of behaviour tests including the Elevated Plus Maze (EPM) and Novel Object Recognition (NOR) for anxiety and memory were performed. acetylcholine levels, acetylcholinesterase (AchE) activity, and M1 receptors were measured by biochemical measures of pre‐frontal cortex (PFC) and hippocampus (HC).

**Result:**

The AD group showed dramatic cognitive declines: there was less novel object searching on the NOR and more anxiety‐like behavior on the EPM. Biochemical measurements demonstrated cholinergic deficiencies: reduced acetylcholine and elevated AchE in the PFC and HC. Treatment with Carum *Copticum* and Thymol produced dramatic changes in both behavioural and biochemical variables, just like Donepezil and Memantine. Notably, Carum *Copticum* and Thymol restored acetylcholine levels and modulated M1 receptor expression, suggesting a role in enhancing cholinergic transmission and providing neuroprotection.

**Conclusion:**

Carum *Copticum* and Thymol show potential as therapeutic agents for AD, demonstrating cognitive and behavioural improvements in an AD model. By modulating acetylcholine levels and AchE activity, these compounds may offer a dual mechanism of neuroprotection and cognitive enhancement. Future studies should investigate these findings in clinical settings, exploring Carum *Copticum* and Thymol as Adjunct or alternative treatments to conventional AD therapies.

